# Sources of perceived social support and cognitive function among older adults: a longitudinal study in rural China

**DOI:** 10.3389/fnagi.2024.1443689

**Published:** 2024-10-09

**Authors:** Shiqi Gui, Jing Wang, Qiushuo Li, Hao Chen, Zhiyue Jiang, Jin Hu, Xing Yang, Jingyuan Yang

**Affiliations:** ^1^The Key Laboratory of Environmental Pollution Monitoring and Disease Control, Department of Epidemiology and Health Statistics, School of Public Health, Guizhou Medical University, Guiyang, China; ^2^The Third People's Hospital of Guizhou Province, Guiyang, China; ^3^School of Medicine and Health Management, Guizhou Medical University, Guiyang, China

**Keywords:** perceived social support, cognitive function, older adults, rural areas, hierarchical linear regression model

## Abstract

**Background:**

Studies have shown the positive impact of perceived social support on cognitive function among older adults in rural areas. However, existing studies often overlook the impact of different support sources. This study aimed to explore the relationship between the diverse sources of perceived social support and cognitive function.

**Methods:**

Participants were drawn from the Guizhou Rural Older Adults’ Health Study (HSRO) in China. We included 791 participants who participated in a baseline survey in 2019 and a 3-year follow-up survey. Perceived social support was investigated from the six main sources (friend, relative, children, spouse, sibling, and neighbor). Hierarchical linear regression models were used to observe the effects of diverse sources of perceived social support and their combinations on cognitive function.

**Results:**

Cognitive function was positively associated with perceived support from children, friends, and neighbors. A positive association was found between cognitive function and increases in each additional source [*β* = 0.75 (95%CI: 0.51, 0.98), *p* < 0.001]. Older adults who perceived support from both children and friends showed better cognitive function [*β* = 2.53 (95%CI: 1.35, 3.72), *p* < 0.001]. The perception of support from spouse, siblings, and relatives did not show a statistically significant association with cognitive function among older adults in rural areas.

**Conclusion:**

This study found that the association between different sources of perceived social support and cognitive function was varied. This study provides scientific evidence that personalized support strategies may benefit in promoting cognitive health in rural older adults.

## 1 Introduction

Cognitive function plays a crucial role in determining the quality of life as a vital dimension of healthy aging (Fang et al., 2015). Cognitive deterioration in older adults causes an array of mental health issues, including delirium, depression, and Alzheimer’s disease (Gauthier et al., 2006; Insel and Badger, 2002). It is estimated that dementia prevalence will triple to 132 million individuals by 2050, highlighting the necessity for interventions aimed at cognitive preservation and dementia prevention to mitigate the challenges to public health and social welfare (World Health Organization, 2021).

Perceived social support refers to the emotional experience and satisfaction that an individual feels respected, supported, and understood within the social network (Kelly et al., 2017; Lu et al., 2019), which has been shown a positive effect on the cognitive function of older adults (Doreste-Mendez et al., 2023; Posis et al., 2023; Kotwal et al., 2016). Although the precise mechanism is unknown, the stress buffer hypothesis could explain this association. In rural areas, older adults suffer from a compounded challenge: lower socioeconomic status, poorer health behaviors, and multiple disease risks (e.g., hypertension, diabetes) — all of which are potential contributors to dementia (Brooks et al., 2023; Dugani et al., 2021; Fu et al., 2023; Ziller and Milkowski, 2020). Perceived social support reduces negative reactions to stressful experiences and negative subjective self-evaluations of cognition, reduces inflammatory responses, contributes to healthy behaviors, and in turn affects cognitive function (Gellert et al., 2018; Ihle et al., 2020; Kuiper et al., 2017; Berkman et al., 2000). With scarce resources in rural areas, the cognitive health of older adults is more susceptible to the protective effects of perceived social support in their social networks (Chen et al., 2024; Harling et al., 2020; Peng et al., 2023). Notably, the effect sizes of perceived social support from diverse sources in a social network on older adults’ cognitive function vary, as well as inevitably being influenced by cultural contexts (Du et al., 2023; Ge et al., 2017; Yin et al., 2020). In addition, different sources of perceived support in social networks may vary in their effects on various aspects of stress buffering (Lee and Goldstein, 2016; Krause, 1986), potentially interacting to influence cognitive health collectively. However, there is limited evidence on whether the diversity of perceived social support (i.e., the accumulation or different combinations of sources of perceived social support) is beneficial to the cognitive health of rural older adults. Therefore, more detailed studies are needed to fill this knowledge gap on the effects of the diversity of sources on cognitive function among older adults in rural areas.

We hypothesized that the sources of perceived social support would affect cognitive function differently among older adults, and further investigated the relationship between distinct patterns of sources (i.e., different combinations of sources) of perceived social support and cognitive function. Using longitudinal data from the Guizhou Rural Older Adults’ Health Study (HSRO) in China, we hope to reveal whether specific types of social support or their unique combination of modalities are critical for maintaining or improving cognitive health in rural older adults.

## 2 Methods

### 2.1 Study design and participants

This was a longitudinal research design with participants from the Guizhou Rural Older Adults’ Health Study (HSRO) in China. The HSRO is a population-based study that employs a multistage cluster sampling approach, selecting participants from 12 villages in Guizhou, China, to ensure a representative sample. Eligible participants were community volunteers who were aged ≥60 years and had been long-term inhabitants of the location (at least >6 months). Data on sociodemographic, healthy behavior, psychology, and healthcare services were collected. The study utilized a longitudinal survey design with a baseline survey T0 (2019) and a follow-up survey T1 (2022), which included 1,795 older adults at baseline and 792 older adults at follow-up. Participants were excluded due to incomplete information on cognitive function. Ultimately, a total of 791 older adults were included in the longitudinal survey. Details about HSRO can be found elsewhere (Chen et al., 2024; Chen et al., 2023). The study was approved by the Ethics Committee of Guizhou Medical University, and all participants signed an informed consent form.

### 2.2 Cognitive function

Cognitive function was consistently assessed by the Chinese version of the Mini-Mental State Examination (MMSE) in surveys (Folstein et al., 1975). The Chinese version of the MMSE is widely used in China for the clinical diagnosis of cognitive impairment and has shown adequate validity and reliability in this population, with an interrater correlation coefficient of 0.998 (Li et al., 1989). The MMSE evaluates several cognitive domains including orientation, episodic memory, language ability, attention and computation, and visual construction. We summarized all cognitive scores to reflect participants’ cognitive function (0–30). Orientation (0–10) involved assessing knowledge of time (day, month, year, season, and the day of the week) and location (state, county, town, or city, building floor, and place name). Immediate (0–3) and delayed (0–3) recall were assessed by memorizing a set of words. Language ability (0–8) contained naming objectives (0–2), following orders (0–3), repeating (0–1), reading (0–1), and writing sentences (0–1). Attention and computational power (0–5) were tested by consecutive subtractions of 100–7 (0–5). Visual construct (0–1) is measured by drawing overlapping pentagons.

### 2.3 Sources of perceived social support

In the HSRO, sources of perceived social support were collected comprehensively in three aspects: In case of an emergency, who do you think would be willing to (1) listen to you; (2) give console and care; (3) help you solve practical problems? Sources include parents, spouse, children, siblings, relatives, friends, neighbors, colleagues, formal official organizations (e.g., government), informal official organizations (e.g., charitable organizations, communities), and other supporters. When participants reported multiple individuals with whom they had the same source, these individuals were treated as one source. Perceived support was assessed from each source, categorizing each source into dichotomous variables: no perceived support and perceived support (at least one aspect is met).

To ensure sufficient statistical power in the statistical analyses of sources of perceived social support, sources with a frequency exceeding 10% (number of participants with perceived support) were selected for further analysis, including friends, siblings, relatives, children, spouse, and neighbor, with details in Supplementary Table S1.

### 2.4 Covariates

The following measurements served as potential covariates: (1) socio-demographic characteristics, including gender, age, economic status (annual household income), marital status, empty nest status, and formal educational level; (2) health-related behaviors, including chronic disease conditions, self-reported health, body mass index (BMI), depression symptoms, and limitations on activities of daily living (ADL).

Information on chronic diseases included hypertension, diabetes, dyslipidemia, heart disease, stroke, lung disease, arthritis or rheumatism, dermatosis, digestive disease, and asthma. Limitations were considered as 1 or more dependencies in ADL, including dressing, eating, housing, walking, and 10 other items (Lawton and Brody, 1969). Depression symptoms were measured by the Patient Health Questionnaire-2 (PHQ-2), with scores of 3 and more defined as positive (Kroenke et al., 2003). Empty nesters are defined as older adults who have no children or have not lived with their children for more than 1 year. The categorization of annual household income is based on the data from the official website of the National Bureau of Statistics: http://www.stats.gov.cn/. The definitions and codes for variables are detailed in Supplementary Table S2.

### 2.5 Statistical analyses

The descriptive statistics were presented with categorical variables shown as proportions, and continuous variables displayed with means and standard deviations (SD). Repeated measures ANOVA is used for longitudinal comparisons between different groups to examine variation in data at different time points.

Further, hierarchical linear regression models (HLM) were conducted to correct the cluster effect of educational level on MMSE assessments among older adults (Hoffman and Walters, 2022; Li et al., 2016). The Intraclass correlation coefficient (ICC) for cognitive function in this study was 0.456, indicating that moderate variability in cognitive function is explained by differences in educational level. Maximum likelihood estimation was used for each model to ensure comparability through fit indices (Luke, 2004), like the Akaike information criterion (AIC). There was a chance that marital status (Yin et al., 2020) and empty nest status (Xu et al., 2023; Zhao et al., 2021) affect both perceived social support and cognitive function, they were included as confounders in the statistical model, and generalized variance inflation factors (GVIFs) were used to check for multicollinearity in the model. Additionally, we conducted a subgroup analysis by gender and age to test the robustness of our results. We found that adding random slopes did not improve the model, therefore, we used a random intercept to ensure the simplicity and accuracy of the model. All statistical analyses were conducted by R version 4.3.3, with a significance level of 0.05.

## 3 Results

### 3.1 Sample characteristics

The study included 791 participants, consisting of 318 men and 473 women, with an average age of 70.67 years (SD = 5.89). The characteristics of the analytical sample are illustrated in Table 1. The average number of years of schooling was 2.76 (SD = 3.08), of which 697 (88.12%) participants had received primary education or less, indicating a generally low level of educational attainment in this cohort. A majority of the older adults, amounting to 62.83%, were in partnered status. There were 26 participants (3.29%) with limitations on ADL and 113 (14.29%) had depressive symptoms. Participants who were older empty nesters were 422 (53.35%). Of all sources, children (70.42%) are the most common source of perceived support for older adults, followed by neighbors (60.56%) and spouse (53.35%). BMI and self-reported health were not statistically associated with changes in MMSE scores at the baseline and follow-up.

**Table 1:** The characteristics of study participants.

Characteristic	*N* (%)	MMSE score, mean (SD)		*F*-test	*p*-value
		Baseline	Follow-up		
Gender				**13.841**	**<0.001**
Male	318(39.82)	24.20(4.31)	22.72(4.40)		
Female	473(60.18)	20.04(4.98)	18.30(4.81)		
Age (years)				**55.152**	**<0.001**
60–69	364(46.02)	22.78(4.80)	21.28(4.43)		
70–79	365(46.14)	21.19(5.11)	19.65(4.95)		
≥80	62(7.84)	18.53(5.52)	15.61(5.12)		
Educational level				**13.938**	**<0.001**
No formal education	315(39.82)	18.37(4.64)	16.33(4.46)		
Primary school	382(48.29)	23.39(4.15)	21.87(4.10)		
Middle school or above	94(11.88)	26.10(3.50)	25.34(2.86)		
Economic status				**22.824**	**<0.001**
Poor	396(50.06)	21.57(5.03)	19.64(4.94)		
So so	215(27.18)	22.47(5.04)	19.94(5.58)		
Rich	180(22.76)	23.17(4.72)	21.22(4.49)		
Marital status				**8.917**	**<0.001**
Partnered	497(62.83)	22.37(4.99)	20.73(5.00)		
Single	294(37.17)	20.48(5.19)	18.98(4.98)		
Chronic disease conditions				1.638	0.807
No	443(56.00)	21.85(4.20)	20.02(5.29)		
Yes	348(44.00)	21.18(4.08)	20.16(4.74)		
BMI (kg/m^2^)				1.621	0.903
Underweight	67(8.47)	21.48(5.30)	19.88(5.33)		
Normal weight	456(57.65)	21.77(5.05)	20.15(5.05)		
Overweight/obesity	268(33.88)	21.67(5.27)	20.01(5.01)		
Limitations on ADL				**15.926**	**<0.001**
No	765(96.73)	21.95(4.96)	20.34(4.84)		
Yes	26(3.29)	14.77(5.55)	12.58(6.42)		
Depressive symptoms				**3.201**	**0.037**
No	678(85.71)	21.92(4.17)	20.18(5.04)		
Yes	113(14.29)	20.46(3.84)	19.45(5.10)		
Self-reported health				2.684	0.185
Unhealthy	524(66.24)	21.55(3.98)	19.90(4.85)		
Healthy	267(33.76)	22.04(5.45)	20.44(5.42)		
Empty nester				**9.323**	**0.003**
No	369(46.65)	21.12(4.29)	19.32(5.09)		
Yes	422(53.35)	22.23(4.96)	20.74(4.94)		
Perceived social support					
From spouse				**19.374**	**<0.001**
No	371(46.90)	20.84(4.15)	18.96(4.90)		
Yes	420(53.10)	22.49(5.02)	21.07(5.02)		
From friends				**11.179**	**<0.001**
No	555(70.16)	21.24(4.10)	19.56(5.00)		
Yes	236(29.84)	23.24(4.98)	21.79(4.91)		
From neighbors				**8.979**	**0.005**
No	312(39.44)	20.79(4.41)	19.48(5.06)		
Yes	479(60.56)	22.13(4.97)	20.36(5.03)		
From children				**10.311**	**0.002**
No	234(29.58)	20.58(4.02)	18.79(5.06)		
Yes	557(70.42)	21.96(5.14)	20.37(5.02)		
Form siblings				**12.382**	**<0.001**
No	586(74.08)	21.38(4.23)	19.56(5.15)		
Yes	205(25.92)	22.67(4.78)	21.58(4.51)		
Form relatives				**10.708**	**0.001**
No	597(75.47)	21.42(4.18)	19.69(4.18)		
Yes	194(24.53)	22.60(4.94)	21.28(3.49)		

Bold values indicate statistically significant differences.

### 3.2 The association between sources of perceived social support and cognitive function

The relationships between sources of perceived social support and cognitive function are shown in Table 2. In Model 1, where each source was estimated independently, perceived support from children (*β* = 1.21, *p* < 0.001, $R_{p}^{2}$ = 0.113), neighbors (*β* = 0.96, *p* < 0.001, $R_{p}^{2}$ = 0.085), and friends (*β* = 0.67, *p* < 0.01, $R_{p}^{2}$ = 0.049) was significantly associated with cognitive function. Perceived support provided by spouses, siblings, or relatives was not significantly related to cognitive function. Except for covariates, all sources of support were controlled simultaneously in Model 2, and the associations of perceived support from children (*β* = 1.17, *p* < 0.001, $R_{p}^{2}$ = 0.106), neighbors (*β* = 0.79, *p* < 0.001, $R_{p}^{2}$ = 0.072), and friends (*β* = 0.49, *p* < 0.05, $R_{p}^{2}$ = 0.044) with cognitive function remained robust (complete results was shown in Supplementary Table S3). Perceived support provided by spouse (*p* = 0.298), siblings (*p* = 0.114), or relatives (*p* = 0.728) was not significantly related to cognitive function. The results of GVIFs showed that there was no high correlation between the independent variables in Supplementary Table S4.

**Table 2:** Associations between the source of perceived social support and cognitive function.

	Model 1						Model 2
Fixed effect	*β*						*β*
Minimally adjusted^a^							
Children	1.31***						1.24***
Neighbors		1.02***					0.81***
Friends			0.71**				0.51*
Spouses				0.37			−0.17
Relatives					0.18		−0.43
Siblings						0.06	0.06
Model statistics							
−2 Log Likelihood	9022.1	9027.6	9041.4	9047.9	9048.3	9050.0	9004.4
AIC	9048.1	9053.6	9067.4	9073.9	9074.3	9076.0	9040.4
Fully adjusted^b^							
Children	1.21***						1.17***
Neighbors		0.96***					0.79***
Friends			0.67**				0.49*
Spouses				0.30			−0.19
Relatives					0.24		−0.37
Siblings						0.05	0.04
Model statistics							
−2 Log Likelihood	9014.2	9017.7	9029.7	9035.8	9036.2	9037.3	8998.2
AIC	9052.2	9055.7	9067.7	9073.8	9074.2	9075.3	9046.2
Observations	1,582						
Number of participants	791						

^a^Gender, age, economic status, marital status, and empty nest status. ^b^Gender, age, economic status, marital status, empty nest status, chronic disease conditions, self-reported health, BMI, depression symptoms, and limitations on ADL. *p ≤ 0.05; **p ≤ 0.01; ***p ≤ 0.001.

### 3.3 The association between combinations of source and cognitive function

We further analyzed the three sources of support (children, friends, and neighbors) that were statistically significant for cognitive function. These three sources of support formed a pattern of eight possible combinations (Supplementary Figure S1). Only 18.08, 7.96, and 3.16% of participants got perceived support from children, neighbors, and friends separately. Approximately 40.58% of participants got perceived support from two sources, while 16.81% of participants got perceived support from all three sources. Table 3 shows the associations between the different source patterns and cognitive function. The result of Model 1 demonstrated a significant positive association between cognitive function and growth in each additional source (as continuous variables) [*β* = 0.75(95%CI: 0.51, 0.98), *p* < 0.001, $R_{p}^{2}$ = 0.067]. Trends between cognitive function and the number of cumulative sources persisted according to age and sex interactions (Supplementary Figure S2). In Model 2, meeting all three sources was significantly associated with superior cognitive function compared to those without support from all three (as categorical variables) [*β* = 2.27 (95%CI:1.48, 3.07), *p* < 0.001]. The strongest effect was observed in the combination of perceived support from both children and friends [*β* = 2.53 (95%CI, 1.35, 3.72), *p* < 0.001].

**Table 3:** Associations between perceived social support provided by child, neighbor, and friend and cognitive function.

	Model 1		Model 2	
Fixed effect	*β* (95%CI)	$R_{p}^{2}$	*β* (95%CI)	$R_{p}^{2}$
(Intercept)	23.69*** (19.32, 28.03)	–	23.40*** (19.03, 27.75)	–
Accumulation of sources	0.75*** (0.51, 0.98)	0.067		
Sources of perceived social support (reference: none)				0.116
Children only			1.03** (0.28, 1.79)	
Friends only			1.56** (0.40, 2.73)	
Neighbors only			1.22** (0.32, 2.12)	
Children + Friends			2.53*** (1.35, 3.72)	
Children + Neighbors			1.98*** (1.29, 2.68)	
Friends + Neighbors			0.49 (−0.87, 1.86)	
Children + Friends + Neighbors			2.27*** (1.48, 3.07)	
Gender (reference: male)	−2.18*** (−2.81, −1.57)	0.224	−2.19*** (−2.82, −1.58)	0.223
Age (reference: 60–69 years)		0.221		0.219
70–79 years	−0.70** (−1.20, −0.20)		−0.70** (−1.20, −0.20)	
≥80 years	−2.54*** (−3.36, −1.72)		−2.54*** (−3.37, −1.73)	
Economic status (reference: poor)		0.109		0.108
So so	0.30 (−0.13, 0.74)		0.28 (−0.16, 0.71)	
Rich	1.19*** (0.65, 1.73)		1.17*** (0.63, 1.70)	
Chronic disease (reference: yes)	0.11 (−0.20, 0.42)	–	0.10 (−0.20, 0.41)	–
Self-reported health (reference: healthy)	−0.11 (−0.37, 0.14)	–	−0.12 (−0.37, 0.14)	–
Normal	−0.12 (−0.90, 0.67)	–	−0.10 (−0.88, 0.69)	–
Overweight	0.43 (−0.42, 1.29)	–	0.46 (−0.39, 1.31)	–
Limitations on ADL (reference: yes)	0.88 (−0.17, 1.82)	–	0.91 (−0.13, 1.85)	–
Depressive symptoms (reference: no)	−0.20** (−0.34, −0.06)	0.022	−0.19** (−0.33, −0.05)	0.015
Empty nesters (reference: no)	0.58* (0.03, 1.14)	0.055	0.60* (0.05, 1.16)	0.054
Marital status (reference: single)	0.12 (−0.41, 0.65)	-	0.10 (−0.43, 0.63)	–
Model statistics				
Observations	1,582			
Number of participants	791			
−2 Log Likelihood	9000.4		8986.5	
AIC	9038.4		9036.5	

*p ≤ 0.05, **p ≤ 0.01, ***p ≤ 0.001; $R_{p}^{2}$ represents the partial *R*^2^, indicating the variable’s explanatory power for the dependent variable after controlling for other variables.

## 4 Discussion

Our hypothesis was supported by the findings which showed an association between perceived social support from diverse sources and cognitive function among rural older adults. Perceived support from children, neighbors, and friends was statistically associated with cognitive function. Furthermore, there was a positive association between cognitive function and growth in each additional source (i.e., there was a cumulative effect). Especially, the strongest effect was observed in the combination of perceived support from both children and friends. However, in the study, perceived social support provided by spouses, siblings, and relatives had no statistically significant relationship with cognitive function.

### 4.1 The association between sources of perceived social support and cognitive function

A study from China showed an association between perceived support from the family on cognitive function (Zhu et al., 2012). However, this study supports the finding that perceived support from children, but not from spouses, was associated with cognitive function in older adults. Traditionally, parents devote resources to raising their children when they are young and expect to receive care and support from their children when they are old (Pan et al., 2017), especially rural people, who have less financial income and are more dependent on their children in their later years. This expectation reflects not only emotional ties within the family but also the transfer of resources and responsibilities that contribute to a supportive family environment, which in turn benefits the older adults’ cognitive function (Khalaila and Litwin, 2011). Additionally, the role of neighbors has been less considered in previous literature. Older adults in rural areas have more neighborhood social capital than in urban communities (Han and Chung, 2022). Closer-knit community ties allow neighbors to build social bonds and help each other (i.e., reciprocity) (Campbell and Lee, 1992; Henning and Lieberg, 1996). This perceived social cohesion may have a positive effect on the cognitive health of older adults. Also, friends who share similar ages and experiences tend to socialize and confide in each other during leisure time as they age, which promotes cognitive stimulation and slows cognitive decline (Fratiglioni et al., 2000; Najar et al., 2019). We did not find a relationship between perceived social support from spouse, relatives, and siblings and cognitive function, possibly due to the inherent complex emotional entanglements and long-standing emotional baggage within these relationships (Khondoker et al., 2017). Additionally, interactions with these social connections may not enhance an individual’s social comparison and self-esteem as effectively as other social relationships. This suggests that social support from different sources may have its own unique nature and effects, indicating that we need to consider these differences more carefully in research and practice.

### 4.2 The association between combinations of source and cognitive function

Furthermore, we explored the association between the combination of perceived social support networks and cognitive function. A robust social support network not only alleviates stress and enhances mental health, but also improves life satisfaction and well-being (Nguyen et al., 2016). Multiple sources ensure the resilience of perceived social support, allowing for alternative sources to maintain social and emotional connections even if support from one party wanes. It emphasizes the importance of building and maintaining a social support network for cognitive health (Ellwardt et al., 2015). Perry et al. (2022) revealed that the proportion of non-relatives in a social network was positively associated with cognitive function. In this study, the accumulation of sources (children, friends, and neighbors) of perceived support correlates positively with cognitive function in the social support networks, suggesting that targeted strategies may be more effective in meeting the diverse social support needs of older adults. Notably, the strongest positive association between cognitive function and any combination of sources observed was perceived support from children and friends. This phenomenon may reflect the fact that support from children and friends may be more responsive to an individual’s needs and preferences. First, children and friends tend to have an in-depth understanding of older adults’ personalities, behaviors, and historical backgrounds and are therefore able to provide more personalized help and care. Second, perceived support from children and friends is essentially based on intimacy and long-term emotional investment. Such deep interaction may contribute to the maintenance of cognitive function (Harling et al., 2020).

### 4.3 Meaning of the study

Although previous studies showed that older adults in rural areas have a higher risk of cognitive impairment in later life (Harling et al., 2020; Harris et al., 2023; Liu et al., 2022), social support serves as a proxy for cognitive reserve and helps maintain cognitive function (Chen et al., 2024), thereby compensating for the decline in cognitive function in older adults in rural areas. The explorations conducted in this study provide valuable insights into understanding the association between diverse sources of perceived social support and cognitive function among older adults in rural areas. The positive effects of perceived social support on cognitive health have been recognized in earlier studies among older adults in rural areas, but often only a holistic concept was considered. Investigating combinations of sources is a novel and integrative perspective that has not yet been considered in the literature. These findings provide a foundation for future research into improving cognitive function in older adults in rural areas. In some non-rural areas, studies may differ regarding the content, form, and effects of social support, and the impact on cognitive health may be different, and older adults may be more dependent on the support of community services and public facilities (Miller, 2011; Wang et al., 2018). A deeper understanding of the mechanisms through which social support works would be beneficial in improving the quality of life of older adults and contributing to the process of healthy aging.

### 4.4 Limitations

Several limitations of this study should be considered. First, this study was conducted in southwest China, which may limit its applicability to other older adult groups. Second, these categorical labels for perceived social support might not accurately reflect the nuanced variations in the level of support experienced by participants. Additionally, MMSE as a measurement tool limited our ability to delve into more detailed analyses like the cognitive domain. Furthermore, the exposure variable for our outcomes was unbalanced with small cases for some categories (e.g., 3.16% of the sample from friends’ support only), which could affect the parameter estimates in these models. Finally, the retention rate of 44.12% in our study may potentially impact the generalizability of our findings and the conclusions drawn from the study, which needs to be addressed in future analyses.

## 5 Conclusion

This study found that the association between different sources of perceived social support and cognitive function was varied. Our findings suggest that three sources of perceived support from children, friends, and neighbors had positive impacts on cognitive function, with accumulative effects. The greatest effect was perceived support from both children and friends. This study provides scientific evidence for personalized support strategies to prevent or delay cognitive deterioration in rural older adults. It would be beneficial for clinicians to harness community resources to bolster perceived support, facilitating a comprehensive approach to the multifaceted requirements of older adults.

## Data Availability

The original contributions presented in the study are included in the article/Supplementary material, further inquiries can be directed to the corresponding author.
