# Supplementary material for: Sources of perceived social support and cognitive function among older adults: a longitudinal study in rural China
Source: Front Aging Neurosci. 2024 Oct 9;16:1443689. doi: 10.3389/fnagi.2024.1443689 (PMC11496072; doi:10.3389/fnagi.2024.1443689)
Supplement: Supplementary file 1 [file Data_Sheet_1.docx]

Supplementary materials

Supplementary eTable 1 Descriptive characteristics of the complete sample of each source of perceived support, with a frequency exceeding 10% (perceived support) was selected for further analysis.

| Measurements at baseline | N (%) | |
| --- | --- | --- |
|  | No perceived support | Perceived support |
| PSS from relative | 597(75.47) | 194(24.53) |
| PSS from spouse | 371(46.90) | 420(53.10) |
| PSS from children | 234(29.58) | 557(70.42) |
| PSS from sibling | 586(74.08) | 205(25.92) |
| PSS from friend | 555(70.16) | 236(29.84) |
| PSS from neighbor | 312(39.44) | 479(60.56) |
| PSS from parent or parents-in-law | 783(98.99) | 8(1.01) |
| PSS from colleague | 791(100.00) | 0(0.00) |
| PSS from the formal official organization | 791(100.00) | 0(0.00) |
| PSS from the informal official organization | 791(100.00) | 0(0.00) |
| PSS from others | 791(100.00) | 0(0.00) |

**Supplementary eTable 2** The definitions and codes for Covariates.

| **Variable** | **Assignment mode** |
| --- | --- |
| Gender | Male = 1; Female =2 |
| Age (years) | 60-69 =1; 70-79 =2; ≥80 = 3 |
| Educational level | No formal education (0 year) =1; Primary school (1-6 years) =2; Middle school or above (≥7 years) = 3 |
| Marital status | Single (divorced/widowed/single) =1;  Partnered (married/partnered) = 2 |
| Economic status | Poor (< 20,000 RMB/year) = 1; So so (20,000 – 40,000 RMB/year) = 2; Rich (≥ 40,000 RMB/year) = 3 |
| Limitations on ADL | No = 0; Yes = 1 |
| Chronic disease conditions | No = 0; Yes= 1 |
| Depression symptoms | No = 0; Yes = 1 |
| BMI (kg/m^2^) | Underweight (< 18.5 kg/m^2^) = 1; normal weight (18.5-24.0 kg/m^2^) = 2, Overweight/obesity (≥ 24.0 kg/m^2^) = 3 |
| Self-reported health | Unhealthy = 1; Healthy = 2 |
| Empty nesters | No = 1; Yes = 1 |

**Supplementary eTable 3** Complete results controlling for all variables on the association between sources of perceived social support and cognitive function.

|  | Model 1 |  | Model 2 |  |
| --- | --- | --- | --- | --- |
| Fixed effect | β (95%CI) | $R_{p}^{2}$ | β (95%CI) | $R_{p}^{2}$ |
| (Intercept) | 23.39*** (19.40,27.37) | - | 23.43*** (19.00, 27.83) | - |
| Gender (reference: male) | -2.20*** (-2.84, -1.59) | 0.201 | -2.22*** (-2.86, -1.61) | 0.202 |
| Age (reference: 60-69 years) |  | 0.225 |  | 0.218 |
| 70-79 years | -0.71** (-1.21, -0.21) |  | -0.69** (-1.20, -0.20) |  |
| ≥80 years | -2.67*** (-3.50, -1.86) |  | -2.53*** (-3.36, -1.72) |  |
| Economic status (reference: poor) |  | 0.110 |  | 0.108 |
| So so | 0.34 (-0.10, 0.78) |  | 0.32 (-0.12, 0.76) |  |
| Rich | 1.21*** (0.68, 1.75) |  | 1.18*** (0.65, 1.72) |  |
| Empty nesters (reference: no) | 0.62 (0.07, 1.18) | - | 0.61* (0.06, 1.16) | 0.052 |
| Marital status (reference: single) | 0.24 (-0.41, 0.89) | - | 0.23 (-0.42, 0.88) | - |
| Chronic disease (reference: yes) |  |  | 0.10 (-0.21, 0.41) | - |
| Self-reported health (reference: healthy) |  |  | -0.11 (-0.36, 0.15) | - |
| BMI (reference: Underweight) |  |  |  | - |
| Normal |  |  | -0.11 (-0.89, 0.68) |  |
| Overweight |  |  | 0.45 (-0.41, 1.30) |  |
| Limitations on ADL (reference: yes) |  |  | 0.86 (-0.18, 1.80) | - |
| Depressive symptoms (reference: no) |  |  | -0.19** (-0.33, -0.05) | 0.013 |
| Source of perceived social support |  |  |  |  |
| Children (reference: no) | 1.24*** (0.71, 1.77) | 0.113 | 1.17*** (0.63, 1.70) | 0.106 |
| Neighbor (reference: no) | 0.82*** (0.39, 1.25) | 0.085 | 0.79*** (0.36, 1.22) | 0.072 |
| Friend (reference: no) | 0.51* (0.03, 0.99) | 0.049 | 0.49* (0.02, 0.96) | 0.044 |
| Spouse (reference: no) | -0.17 (-0.76, 0.42) | - | -0.19 (-0.77, 0.41) | - |
| Sibling (reference: no) | -0.43 (-0.99,0.13) | - | -0.37 (-0.93, 0.18) | - |
| Relative (reference: no) | -0.06 (-0.50, 0.63) | - | 0.04 (-0.52, 0.60) | - |

*Note:* **p* ≤ 0.05; ***p* ≤ 0.01; ****p* ≤ 0.001; $R_{p}^{2}$represents the partial *R^2^*, indicating the variable's explanatory power for the dependent variable after controlling for other variables.

**Supplementary eTable 4** GVIFs were used to check for multicollinearity between independent variables and cognitive function.

|  | GVIF | Df | Adjusted GVIF |
| --- | --- | --- | --- |
| Gender | 1.056 | 1 | 1.028 |
| Age | 1.075 | 2 | 1.018 |
| Economic status | 1.046 | 2 | 1.011 |
| Empty nester | 1.041 | 1 | 1.020 |
| Marital status | 1.653 | 1 | 1.286 |
| chronic disease | 1.075 | 1 | 1.037 |
| self-reported health | 1.122 | 1 | 1.059 |
| BMI | 1.050 | 2 | 1.012 |
| limitations on ADL | 1.029 | 1 | 1.015 |
| Depressive symptoms | 1.103 | 1 | 1.050 |
| Source: children | 1.695 | 1 | 1.302 |
| Source: neighbor | 1.680 | 1 | 1.296 |
| Source: friend | 1.269 | 1 | 1.126 |
| Source: spouse | 1.036 | 1 | 1.018 |
| Source: sibling | 1.096 | 1 | 1.047 |
| Source: relative | 1.774 | 1 | 1.332 |

*Note:* A high degree of covariance problem exists for GVIF values of the independent variables exceeding 10. Adjusted GVIF values above 1.5 are considered to have some degree of covariance problem. GVIF: generalized variance inflation factor; Df: Degrees of Freedom; BMI: body mass index; ADL: activities of daily living; Adjusted GVIF: GVIF^(1/(2*Df)).


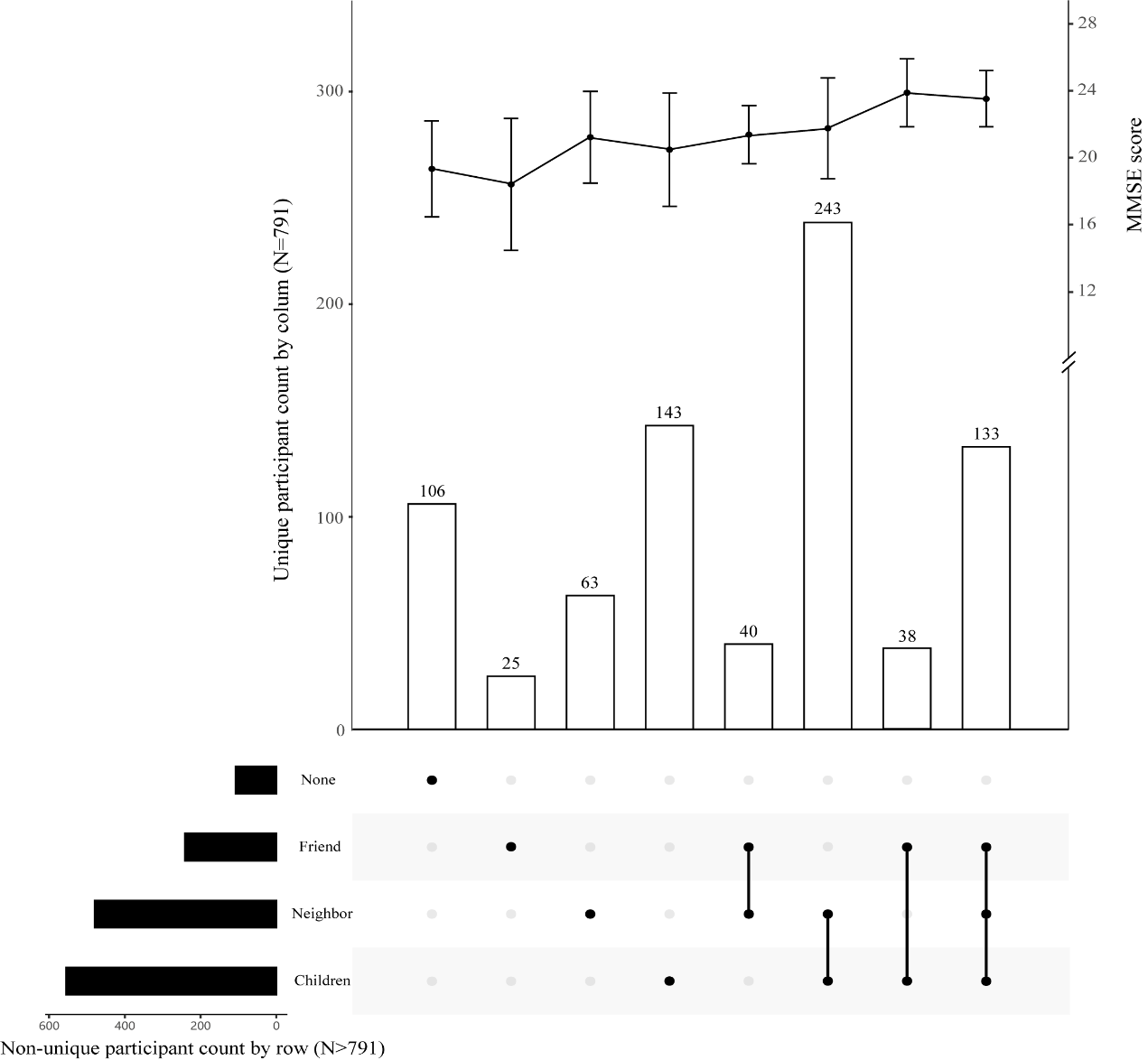
 Supplementary eFigure 1 Distribution of participants with one source of perceived support, multiple sources of perceived support, or all sources of perceived support at baseline. The vertical bars represent the distribution of participants in each of the eight source patterns in this sample. The horizontal bars represent the distribution of participants with a specific source. The folded line represents the trend of MMSE scores (represented by the mean and standard deviation) for each of the eight source patterns in that sample.


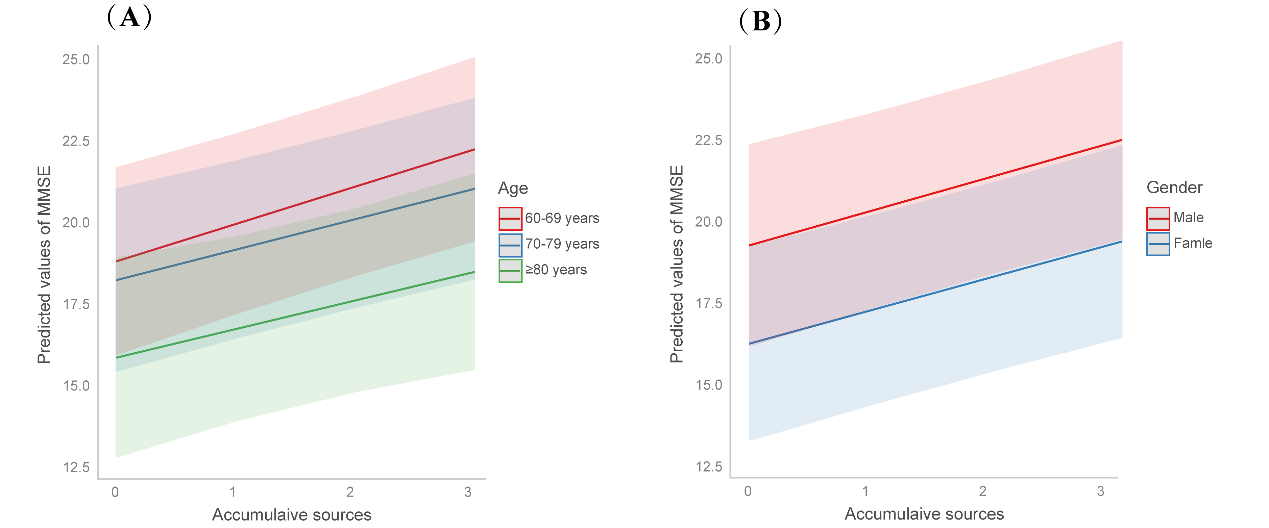


Supplementary eFigure 2 Predicted trajectories of MMSE score by age(A) and gender(B); trajectories represent predicted marginal means for MMSE score over the number of accumulative sources (children, neighbors, and friends), across age and gender. Models are adjusted for economic status, marital status, empty nest status, chronic disease conditions, self-reported health, BMI, depression symptoms, and limitations on ADL. Both models include random intercepts. MMSE: the Mini-Mental State Examination.
